# Proviral functions of HMGB1 in HAdV-C5 replication compartments

**DOI:** 10.1128/spectrum.01958-25

**Published:** 2025-09-08

**Authors:** Paloma Hidalgo, Britta Gornott, Luca D. Bertzbach, Thomas Dobner

**Affiliations:** 1 Department of Viral Transformation, Leibniz Institute of Virology (LIV), Martinistraße, Hamburg, Germany; 2 Research Unit Emerging Viruses, Leibniz Institute of Virology (LIV), Martinistraße, Hamburg, Germany; University of Manitoba, Winnipeg, Manitoba, Canada

**Keywords:** replication compartments, HMGB1, histone chaperone, human adenovirus (HAdV), viral replication

## Abstract

Human adenoviruses (HAdVs) induce significant reorganization of the nuclear environment, leading to the formation of virus-induced subnuclear structures known as replication compartments (RCs). Within these RCs, viral genome replication, gene expression, and modulation of cellular antiviral responses are tightly coordinated, making them valuable models for studying virus-host interactions. In a recent study, we analyzed the protein composition of HAdV type 5 (HAdV-C5) RCs isolated from infected primary cells at different time points during infection using quantitative proteomics. We identified several chromatin modifiers, including the high-mobility group box 1 protein (HMGB1) as components associated with RCs and demonstrated that HMGB1 can be relocalized to RCs from different HAdV species, thereby modulating viral replication in a species-specific manner. In the present work, using click-chemistry and proximity ligation assays, we discovered that HMGB1 localizes to sites of DNA replication within RCs and that its interaction with DBP in RCs is dependent on both DNA replication and RC assembly. HMGB1-knockdown experiments demonstrated that HMGB1 is required for efficient viral gene expression. However, despite its proviral role in viral replication, we found that HMGB1 levels decreased in late stages of infection due to transcriptional downregulation. Furthermore, by overexpressing HMGB1, we showed that this regulation of HMGB1 levels during infection is critical for optimal HAdV-C5 replication. These results highlight the complex regulatory relationship between HMGB1 and HAdV-C5 infection.

**IMPORTANCE:**

In an extensive proteomics analysis, we found that HMGB1, an important cellular chromatin protein, was enriched in adenovirus replication compartments. In this study, we aimed to better understand the role of HMGB1 in the infection process of a human DNA virus, HAdV-C5. We tested different virus types, including some with specific gene deletions and mutations. Our results showed that during infection, HMGB1 levels decreased because the virus suppressed its production. Despite this, even at lower levels, HMGB1 still helped the virus replicate by interacting with key viral proteins and DNA at sites where the virus is actively replicating. Overall, our findings highlight how HMGB1 plays a crucial role in facilitating efficient virus replication, making it an important factor in the infection process.

## INTRODUCTION

Human adenoviruses (HAdVs) are medium-sized viruses with a linear, double-stranded DNA genome that have taught us much about DNA replication, RNA splicing, and cellular biology (1). HAdVs replicate in the nuclei of infected cells where they form so-called viral replication compartments (RCs), which are spherical biomolecular condensates likely formed through liquid-liquid phase separation (2, 3). HAdV-induced RCs facilitate efficient viral replication by serving as a scaffold to concentrate proviral factors necessary for viral DNA replication, late gene expression, and virus assembly. Moreover, host restriction factors can also be localized to RCs, blocking their antiviral functions or repurposing their activities to favor viral replication (2, 4–6).

To elucidate the protein composition of RCs throughout infection, we recently reported a proteomic profile of isolated HAdV-C5 RCs. In this study, we identified several significantly enriched viral and cellular proteins, among them the high-mobility group box 1 (HMGB1) protein (7–9). HMGB1 is a multifunctional protein that belongs to the HMGB domain proteins, essential nuclear components involved in DNA binding, chromatin remodeling, and gene regulation. Among them, HMGB1 and HMGB2 share a high degree of structural and amino acid sequence similarity, with the primary distinction being the length of their C-terminal regions (10). Extensive research has explored the nuclear and extranuclear functions of HMGB1, leading to a substantial body of data on its roles. In contrast, the functions of HMGB2, particularly in the cytoplasm, on the membrane, and in the extracellular space, remain largely unexamined (11, 12). HMGB1 is primarily a nuclear protein; however, depending on its post-translational modifications, it can also localize to the cytoplasm, be actively secreted, or be passively released from cells (13, 14). HMGB1 takes over key roles in intracellular responses to stress, during autophagy and apoptosis, and in intranuclear processes such as DNA replication, recombination, repair, transcription, as well as in maintaining nucleosome integrity (15). In the extracellular space, HMGB1 can function as a proinflammatory cytokine and damage-associated molecular pattern molecule, activating the immune response (13, 14).

Increasing evidence has demonstrated that HMGB1 plays important roles in the replication cycles of several viruses, including HAdVs (16). During HAdV-C5 infection, HMGB1, together with HMGB2, enhances the activation of the major late promoter by the major late transcription factor (MLTF) (17). However, HMGB1 also triggers a proinflammatory response, which is suppressed by the viral protein pVII (18–20) and the virus-associated RNAI (VA RNAI) (21), inhibiting its secretion (22).

Here, we characterized the relocalization of HMGB1 into HAdV-C5 RCs and investigated its role during progression of the infection. Our data show that HAdV-C5 infection induced a reduction of HMGB1 protein levels due to downregulation of the HMGB1 promoter. However, these low levels of HMGB1 enhanced viral replication by binding to DBP (a central protein of HAdV RCs [23]) and localizing to sites of viral DNA replication within RCs.

## RESULTS

### HMGB1 binds to DBP and localizes to sites of DNA replication within RCs

**Fig 1:**
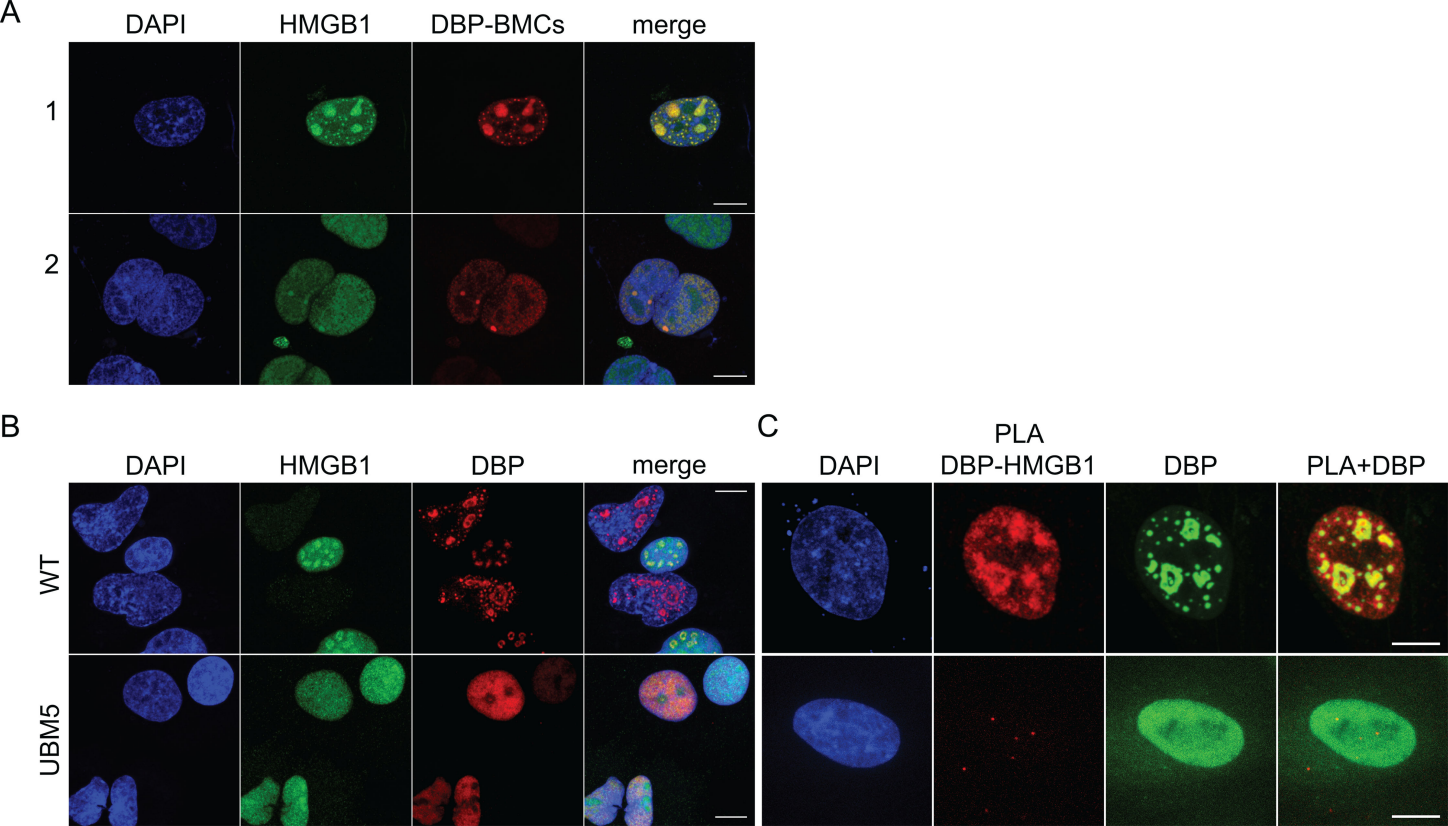
HMGB1 can be relocalized to RCs and DBP-BMCs by binding to DBP. (**A**) H1299 cells were transfected with a plasmid expressing mCherry-DBP (red). The cells were fixed 24 h post-transfection, immunostained for HMGB1 (green). (**B**) A549 cells were infected with HAdV-C5 WT or UBM5 and fixed 24 hpi. The cells were co-immunostained against HMGB1 (green) and DBP (red). (**C**) A549 cells were infected with HAdV-C5 WT or UBM5 and fixed at 24 hpi. The cells were subjected to proximity ligation assays (PLA, red) using primary antibodies against HMGB1 and DBP. Immunostaining for DBP (green) was included as a reference for RCs. PLA DBP-HMGB1: PLA signal for the interaction between DBP and HMGB1. All samples were visualized by confocal microscopy as z-stacks, and the images are presented as maximum-intensity projections. DAPI (blue) was used to visualize DNA. The scale bars correspond to 10 µm.

In order to understand the role of HMGB1 on viral RCs, we used wild-type (WT) HAdV-C5, as well as the DBP mutant, H5pm4251 (UBM5), which consistently lacks RCs (24). Previously, we demonstrated that HMGB1 can be relocalized to RCs (7). HMGB1 even colocalizes with DBP in biomolecular condensates (BMCs) formed during the transient expression of DBP (Fig. 1A). When RCs are not formed during UBM5 infection, HMGB1 remains diffused in the nucleus of infected cells and does not bind to the mutant DBP, as confirmed with the proximity ligation assays (PLAs) (Fig. 1B and C).

**Fig 2:**
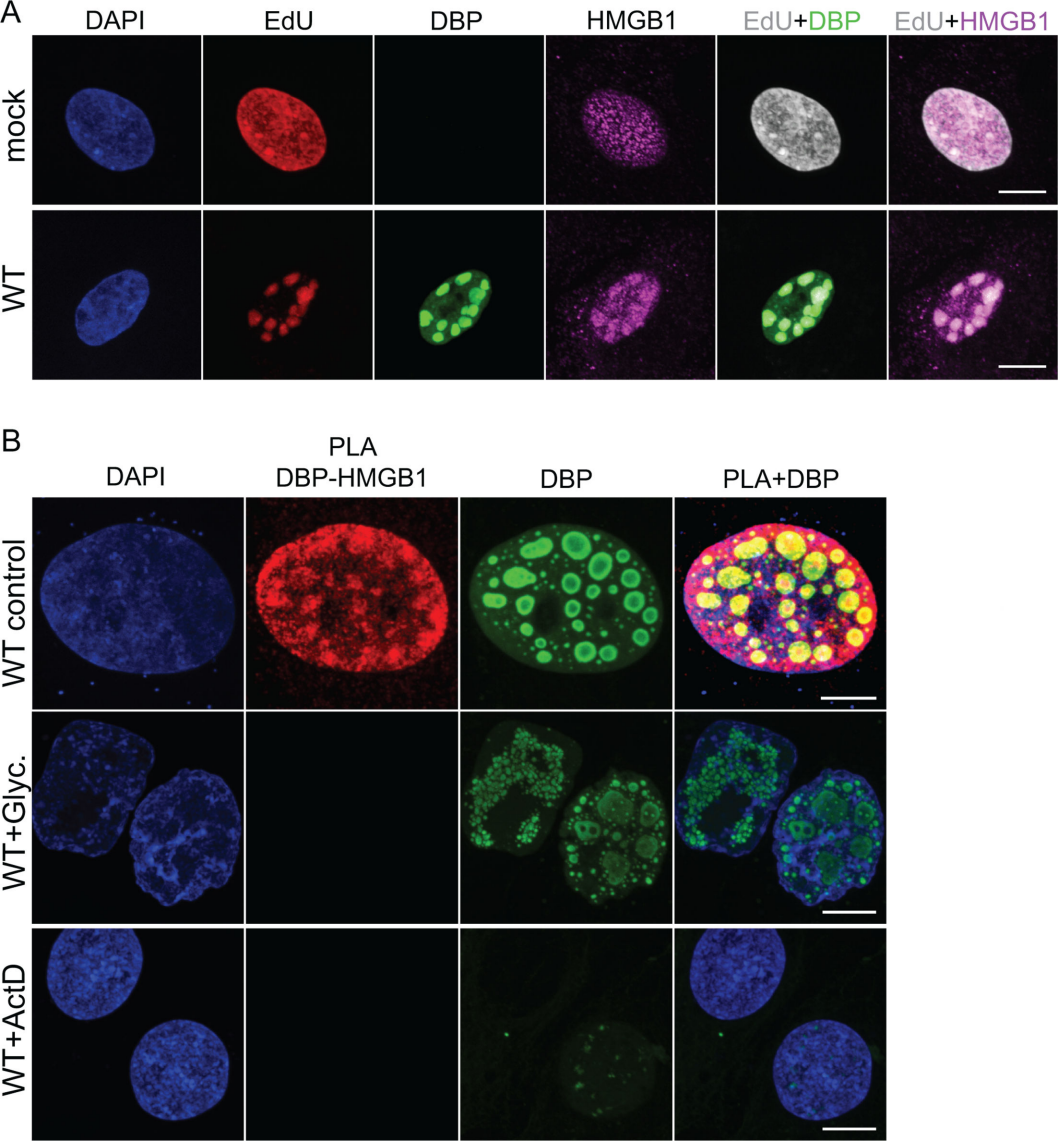
HMGB1 binds to DBP and localizes to DNA replication regions within RCs. (**A**) A549 cells were mock-infected or infected with HAdV-C5 WT, incubated with EdU at 16 hpi, and fixed 24 hpi later. A copper (I)-catalyzed click reaction was performed to conjugate EdU with Alexa Fluor 555 dye (red) to stain the sites of accumulation of newly replicated DNA. Cells were additionally immunostained to detect DBP (green) and HMGB1 (magenta). Merged images of EdU (gray) and DBP (green) or EdU (gray) and HMGB1 (magenta) are included. (**B**) A549 cells were infected with HAdV-C5 WT and fixed at 24 hpi. The cells were subjected to proximity ligation assays (PLA, red) using primary antibodies against HMGB1 and DBP. Immunostaining for DBP (green) was included as a reference for RCs; 4 mM glycyrrhizin (Glyc.) treatment was included to impede HMGB1 interaction with DNA; 100 ng/mL actinomycin D (ActD) treatment was included to inhibit DNA replication. (**A and B**) All samples were visualized by confocal microscopy as z-stacks, and the images are presented as maximum-intensity projections. DAPI (blue) was used to visualize DNA. The scale bars correspond to 10 µm. PLA DBP-HMGB1: PLA signal for the interaction between DBP and HMGB1.

Our previous data established that HMGB1 interacts with DBP and enhances viral DNA synthesis (7). To further investigate this interaction, we assessed whether HMGB1 binds to DBP and viral DNA within RCs. EdU click-chemistry assays demonstrated that HMGB1 colocalizes with newly synthesized DNA within RCs, providing further support for its role in facilitating viral genome amplification (Fig. 2A). HMGB1 has two HMG-boxes, A and B. These boxes are responsible for HMGB1-binding to DNA. Glycyrrhizin binds to HMG-box A and B of HMGB1 and reduces binding of the protein to DNA (25, 26). PLAs, combined with glycyrrhizin treatment, confirmed that the interaction between HMGB1 and DBP depends on HMGB1’s DNA-binding activity (Fig. 2B). Additionally, PLAs in the presence of actinomycin D showed that RCs and HMGB1-DBP binding are disrupted, indicating that DNA replication and RC assembly are required for this interaction (Fig. 2B). This observation is in agreement with the lack of interaction between DBP and HMGB1 in UBM5 infection, where RCs are not formed (Fig. 1). Furthermore, electrophoretic mobility shift assays suggest that HMGB1 may bind to viral DNA (data not shown). Chromatin immunoprecipitation assays should ultimately confirm if HMGB1 binds viral DNA. Altogether, our data indicate that the role of HMGB1 in viral replication and its localization in RCs is mediated through interaction with DBP and localization to sites of DNA replication.

### HMGB1 is required for efficient viral gene expression

**Fig 3:**
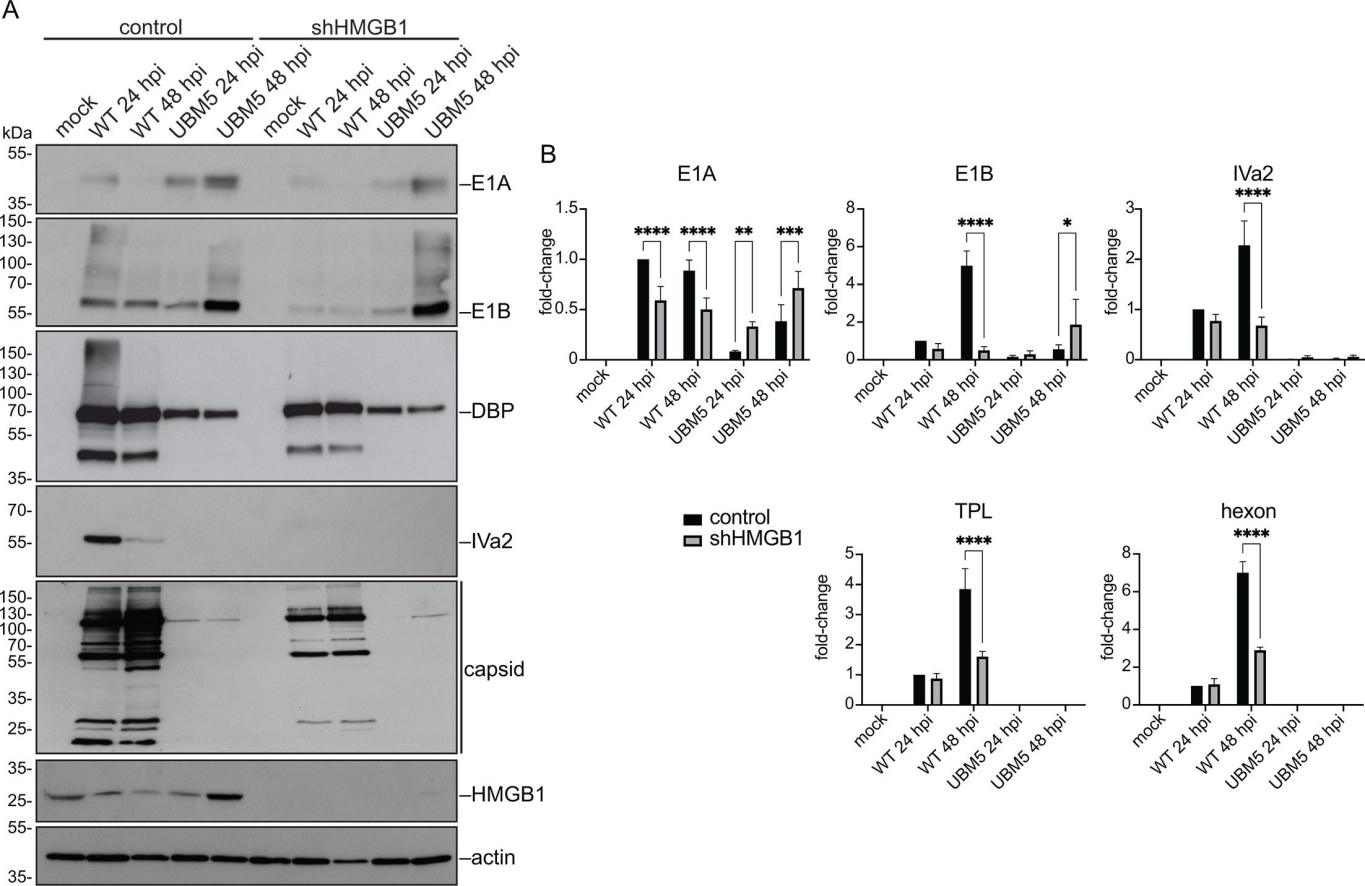
HMGB1 is necessary for efficient viral gene expression. (**A**) To determine whether the absence of HMGB1 affected viral gene expression, viral protein levels were measured by immunoblotting at 24 and 48 hpi, in mock-infected, WT-, or UBM5-infected A549 cells, stably expressing the shRNA against HMGB1 (shHMGB1). (**B**) To measure the steady-state levels of viral mRNA, total RNA was isolated from A549 cells mock-infected or infected with HAdV-C5 WT or UBM5, harvested at 24 or 48 hpi. The RNA was reverse-transcribed and quantified by RT-qPCR using primers for viral early (E1A and E1B), intermediate (IVa2), and late (TPL and hexon) mRNAs. The plots show the fold-change values of the specified mRNAs, normalized to GAPDH mRNA and relative to the WT at 24 hpi in control cells. The error bars represent standard deviations. Data were analyzed using a two-way ANOVA test. *, *P* < 0.05; **, *P* < 0.01; ***, *P* < 0.001, ****, *P* < 0.0001.

Since HMGB1 plays a critical role in viral DNA synthesis and promotes efficient progeny production (7), in addition to localizing with viral DNA within RCs (Fig. 2), we next examined if it could also alter viral gene expression. Knockdown of HMGB1 using shRNAs significantly reduced viral protein expression, particularly of late proteins (Fig. 3A). This reduction in protein expression is accompanied by a decrease in viral mRNA levels (Fig. 3B). Silencing HMGB1 did not restore levels of viral late proteins in UBM5 infection, and early proteins were not significantly altered (Fig. 3A); however, it increased viral early mRNAs (Fig. 3B). In contrast, although HMGB2 is also relocalized to RCs and interacts with DBP, knockdown of HMGB2 does not significantly affect viral protein levels and virus yield (data not shown), indicating that HMGB1 assumes a more specific and crucial role in viral gene expression.

### HMGB1 is downregulated in HAdV-C5 infection

**Fig 4:**
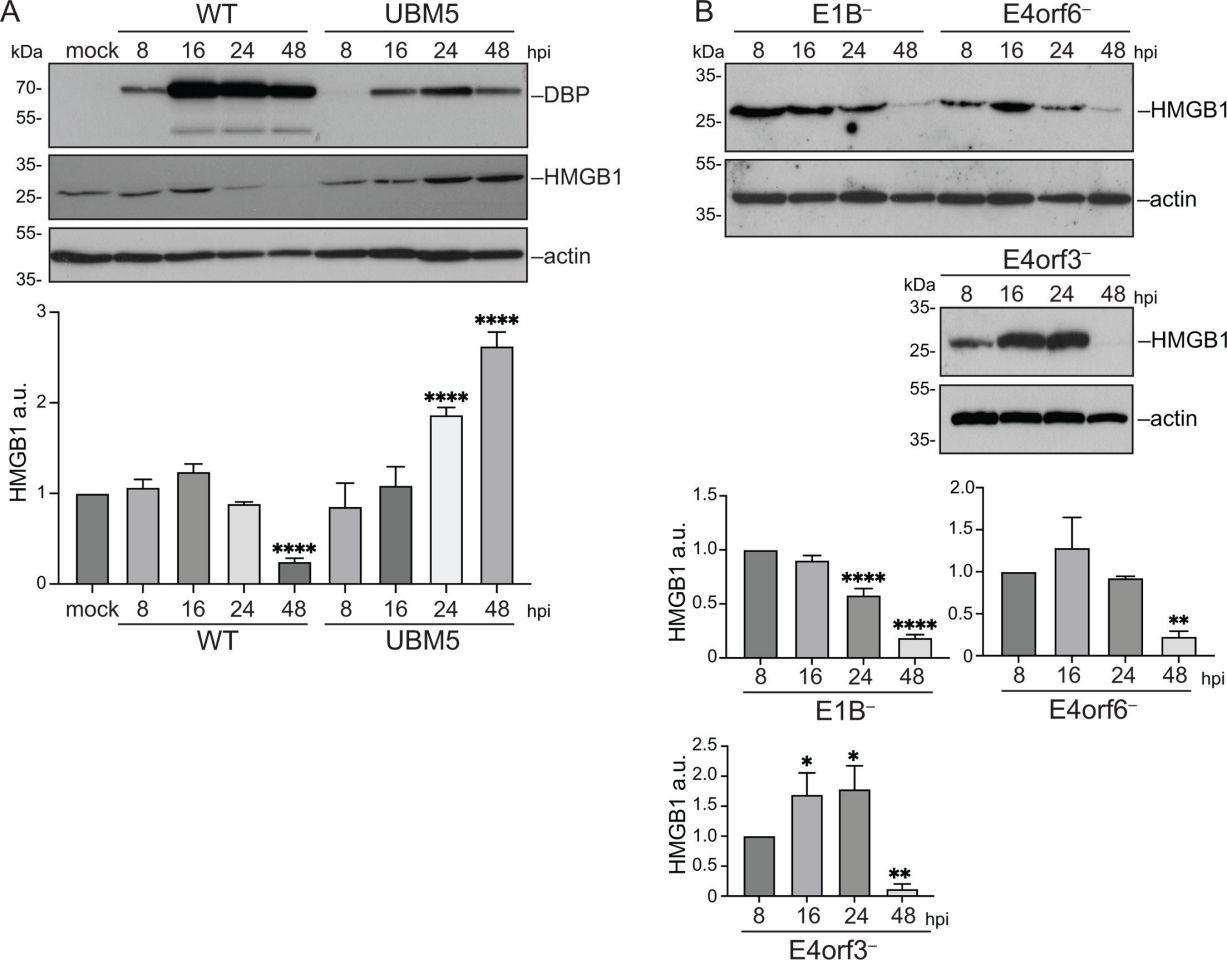
HMGB1 is downregulated in HAdV-C5 infection. (**A**) Steady-state levels of HMGB1 were measured in whole-cell lysates of A549 mock-infected or infected with HAdV-C5 WT or UBM5, harvested at 8, 16, 24, and 48 hpi, and analyzed by immunoblotting. DBP served as a control of the infection, and actin was used as a loading control. The plot shows the average densitometry measurements of HMGB1 from three biological replicates, normalized to actin. The samples were compared relative to the mock-infected cells. The error bars represent standard deviations. Data were analyzed using an ordinary one-way ANOVA test. ****, *P* < 0.0001. (**B**) To evaluate if viral proteins are responsible for downregulation of HMGB1, A549 cells were infected with the E1B−, E4orf6−, or E4orf3− mutant viruses and harvested at 8, 16, 24, and 48 hpi. The protein lysates were analyzed by immunoblotting. Actin was used as loading control. The plots show the average densitometry measurements of HMGB1 from three biological replicates, normalized to actin. The samples were compared relative to 8 hpi for each mutant virus. The error bars represent standard deviations. Data were analyzed using an ordinary one-way ANOVA test. *, *P* < 0.05; **, *P* < 0.01; ****, *P* < 0.0001.

When we compared the intracellular steady-state levels of HMGB1 in WT and UBM5 infection, we observed that, while HMGB1 levels decline at 48 hours post-infection (hpi) in WT infection, they remain stable or even increase in UBM5-infected cells (Fig. 4A). It is noteworthy to mention that cells and infections were not synchronized. Therefore, at the single-cell level, we observed a mixture of infected cells—some with detectable signal for HMGB1 and some with reduced or no HMGB1 staining (Fig. 1B). The heterogeneity observed at the single-cell level may represent a biologically important mechanism that would be interesting to study in future work. However, the average phenotype measured in whole-cell lysates used for immunoblots reflects a general downregulation of HMGB1 at 48 hpi (Fig. 4A). These findings suggest that HMGB1 regulation during infection may depend on both the stage in which the cells were infected and the progression of the viral replication cycle.

This phenotype prompted us to investigate the underlying mechanisms contributing to this reduction. The steady-state level of HMGB1 could be affected by different mechanisms, like protein degradation, changes in its stability, secretion, or transcriptional regulation. Viral proteins like E1B-55K (E1B), E4orf6, and E4orf3 can assemble protein complexes that target cellular proteins for SUMOylation or ubiquitination that can result in proteasome degradation of the modified protein (27–30). Therefore, we tested whether these viral proteins were responsible for the downregulation of HMGB1 at late times post-infection. Western blot analysis of cells infected with E1B-, E4orf6-, or E4orf3-deficient viruses showed that, despite differences in the accumulation patterns at earlier time points compared to WT, HMGB1 levels consistently decrease at 48 hpi across all mutants (Fig. 4B). This indicates that these viral proteins are not directly responsible for this phenotype.

### HMGB1 is transcriptionally repressed in HAdV-C5 infection

**Fig 5:**
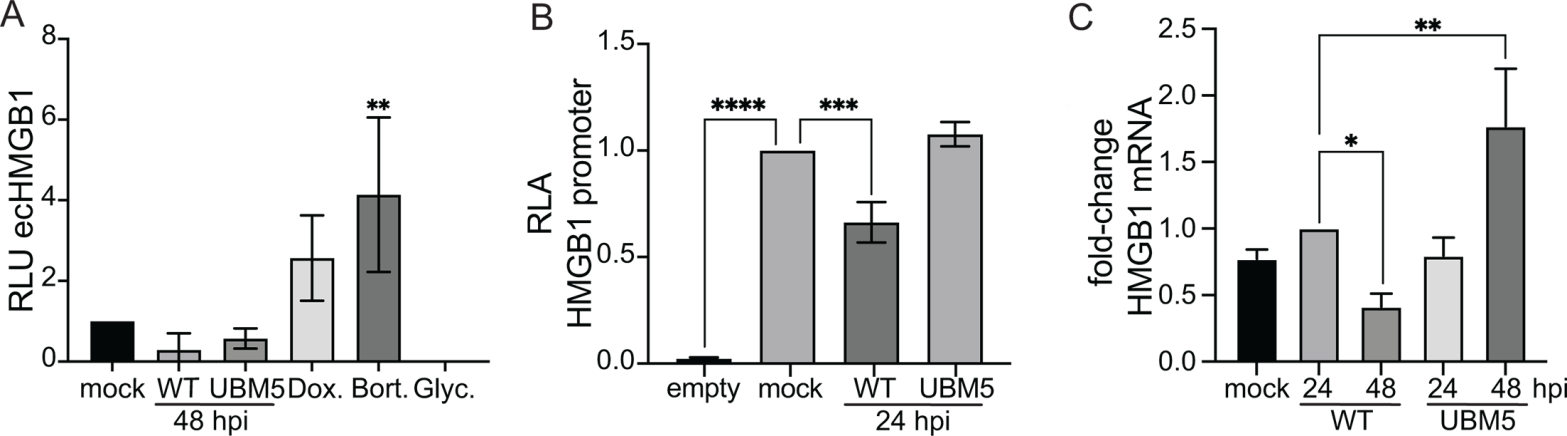
HMGB1 is downregulated during HAdV-C5 infection by transcriptional repression. (**A**) To measure extracellular (ec) HMGB1, the Lumit HMGB1 (Human/Mouse) Immunoassay was used on the supernatant of A549 cells mock-infected or infected with HAdV-C5 WT or UBM5 48 hpi. As positive controls, mock-infected cells were treated with 1 µM doxorubicin (Dox.) or 1 µM bortezomib (Bort.) to induce HMGB1 secretion. As a negative control, mock-infected cells were treated with 4 mM glycyrrhizin (Glyc.) to block HMGB1 secretion. Data are presented as relative luciferase units (RLU). The error bars represent standard deviations. Data were analyzed using an ordinary one-way ANOVA test. **, *P* < 0.01. (**B**) The activity of the HMGB1 promoter was evaluated in H1299 cells co-transfected with the HMGB1 promoter cloned into the pGL3-basic vector and mock-infected or infected with HAdV-C5 WT or UBM5. Cells were analyzed for luciferase activity at 24 hpi. The firefly luciferase was normalized against the renilla luciferase signal to yield the relative luciferase activity (RLA). The error bars represent standard deviations. Data were analyzed using an ordinary one-way ANOVA test. ***, *P* < 0.001; ****, *P* < 0.0001. (**C**) To measure the steady-state levels of the HMGB1 mRNA, total RNA was isolated from A549 cells mock-infected or infected with HAdV-C5 WT or UBM5, harvested at 24 or 48 hpi. The RNA was reverse-transcribed and quantified by RT-qPCR using the ∆∆Ct method. The plots show the fold-change values of the HMGB1 mRNA, normalized to GAPDH mRNA and compared relative to the WT at 24 hpi. The error bars represent standard deviations. Data were analyzed using an ordinary one-way ANOVA test. *, *P* < 0.05; **, *P* < 0.01.

We next investigated HMGB1 secretion and its transcriptional regulation to better understand the underlying cause of its downregulation. The Lumit Immunoassay is a bioluminescent assay for measuring HMGB1 directly in the cell culture medium. This assay revealed no detectable extracellular levels of HMGB1 in infected A549 cells, with positive controls (doxorubicin and bortezomib) confirming the assay’s sensitivity (Fig. 5A). These results indicate that the decrease of HMGB1 levels at 48 hpi is not due to secretion from infected cells. However, luciferase reporter assays of the HMGB1 promoter showed that activation of the HMGB1 promoter was decreased during WT infection but not in UBM5 infection (Fig. 5B). Lower levels of HMGB1 mRNA, measured by RT-qPCR, were observed 48 hpi in WT infection, while in UBM5 infection, the levels increased twofold (Fig. 5C), correlating with HMGB1 protein levels (Fig. 4A). These results indicate that at least transcriptional repression accounts for HMGB1 downregulation during HAdV-C5 late times post-infection.

### Basal or reduced levels of HMGB1 enhance viral replication

Given the proviral effect of HMGB1 on viral infection (7), its downregulation was unexpected. However, this phenotype has already been reported for other proteins, like p53, which—despite promoting viral gene expression and DNA replication—also shows reduced protein levels as infection progresses, as discussed further in the *Discussion* section. Based on this, we hypothesized that reduced levels of HMGB1 actively support viral replication and that overexpression of HMGB1, in turn, impairs it.

**Fig 6:**
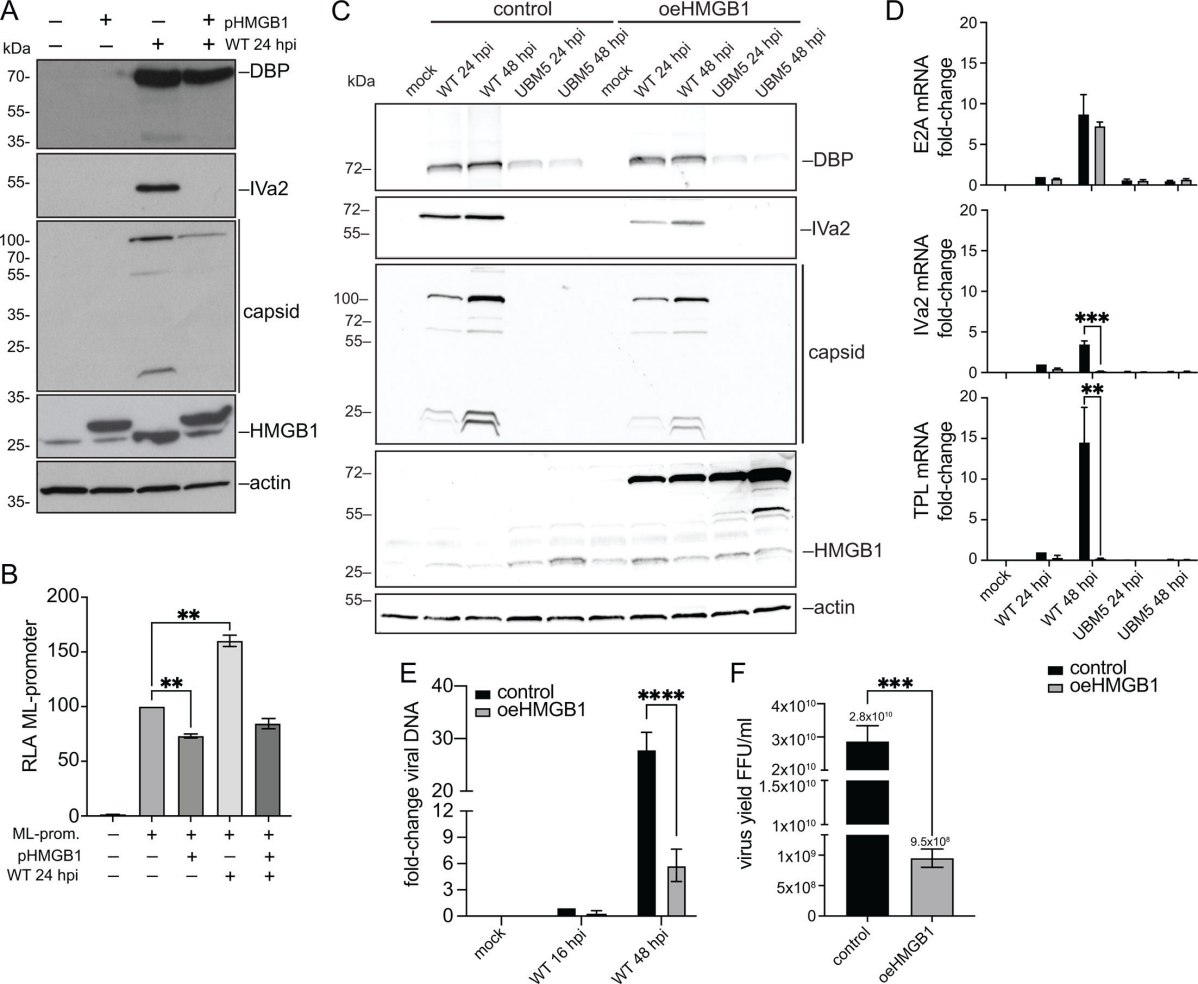
Basal or lower levels of HMGB1 promote viral replication. (**A**) To determine if exogenous and overexpressed levels of HMGB1 affected viral replication, viral protein levels were initially measured by immunoblotting at 24 hpi, in mock-infected or WT-infected H1299 cells, transiently transfected with a vector expressing a Flag-tagged HMGB1 (pHMGB1). HMGB1 was detected using an antibody specific for this protein (Table 1). (**B**) The activity of the major late (ML) promoter (prom.) was evaluated in shHMGB1 H1299 cells, in the absence or presence of HMGB1 and in mock-infected or HAdV-C5 WT-infected cells. Cells were analyzed for luciferase activity at 24 hpi. The firefly luciferase was normalized against the renilla luciferase signal to yield the relative luciferase activity (RLA). The error bars represent standard deviations. Data were analyzed using an ordinary one-way ANOVA test. **, *P* < 0.01. A549 control cells and cells stably overexpressing HMGB1 (oeHMGB1) were prepared to determine whether increased levels of HMGB1 affected viral replication (**C–F**). (**C**) Viral protein levels were measured by immunoblotting at 24 and 48 hpi, in mock-infected, WT-, or UBM5-infected cells. (**D**) To measure the steady-state levels of viral mRNA, total RNA was isolated from cells that were either mock-infected or infected with HAdV-C5 WT or UBM5, harvested at 24 or 48 hpi. The RNA was reverse-transcribed and quantified by RT-qPCR using primers for viral early (E2A), intermediate (IVa2), and late (TPL) mRNAs. The plots show the fold-change values of the specified mRNAs, normalized to GAPDH mRNA and relative to the WT at 24 hpi in control cells. The error bars represent standard deviations. Data were analyzed using a two-way ANOVA test. **, *P* < 0.01; ***, *P* < 0.001. (**E**) HAdV-C5 DNA levels were measured by qPCR at 16 and 48 hpi. The error bars represent standard deviations. Data were analyzed using a two-way ANOVA test. ****, *P* < 0.0001. (**F**) Virus yield was determined using quantitative DBP immunofluorescence. The samples were collected from either control or oeHMGB1 A549 cells, which were infected with HAdV-C5 WT and harvested 48 hpi. The error bars represent standard deviations. Data were analyzed using a two-tailed unpaired t test. ***, *P* < 0.001.

To investigate the functional consequences of HMGB1 overexpression, we observed several key outcomes. First, we transiently transfected cells with a plasmid that overexpresses HMGB1 (pHMGB1) and observed that exogenous overexpression of HMGB1 led to reduced levels of viral late proteins (Fig. 6A). As noted in the introduction, HMGB1 promotes the activation of the major late promoter through MLTF (17). To determine whether the overexpression of HMGB1 had an upstream effect on viral protein expression, we performed luciferase reporter assays to measure the activation of the major late promoter (ML-prom.). For this purpose, we used H1299 cells with HMGB1 knocked down (as described in the Materials and Methods) and transiently overexpressed HMGB1 via pHMGB1 transfection. We evaluated the activity of the ML-prom. in mock-infected and WT-infected cells at 24 hpi (Fig. 6B). The results indicated that exogenous overexpression of HMGB1 represses ML promoter activity (Fig. 6B).

To further assess the impact of HMGB1 overexpression on viral replication, we measured viral proteins (Fig. 6C), viral mRNA (Fig. 6D), viral DNA levels (Fig. 6E), and virus yield (Fig. 6F) in A549 cells stably overexpressing HMGB1 (oeHMGB1). The blots in Fig. 6C show reduced levels of IVa2 and capsid proteins upon HMGB1 overexpression. Similarly, the quantification of viral mRNA indicates impaired viral gene expression when HMGB1 is overexpressed, especially for transcripts expressed late during infection, including IVa2 and the late mRNA containing the tripartite leader (TPL) (Fig. 6D). The qPCR data revealed a reduction of around 80% in viral DNA levels (Fig. 6E), and virus progeny production was also reduced by over one log (Fig. 6F), confirming that HMGB1 overexpression impairs viral replication. Together, these results indicate that precise regulation of HMGB1 levels is critical for an efficient viral replication cycle.

### Downregulation of HMGB1 during HAdV infection is species-specific

**Fig 7:**
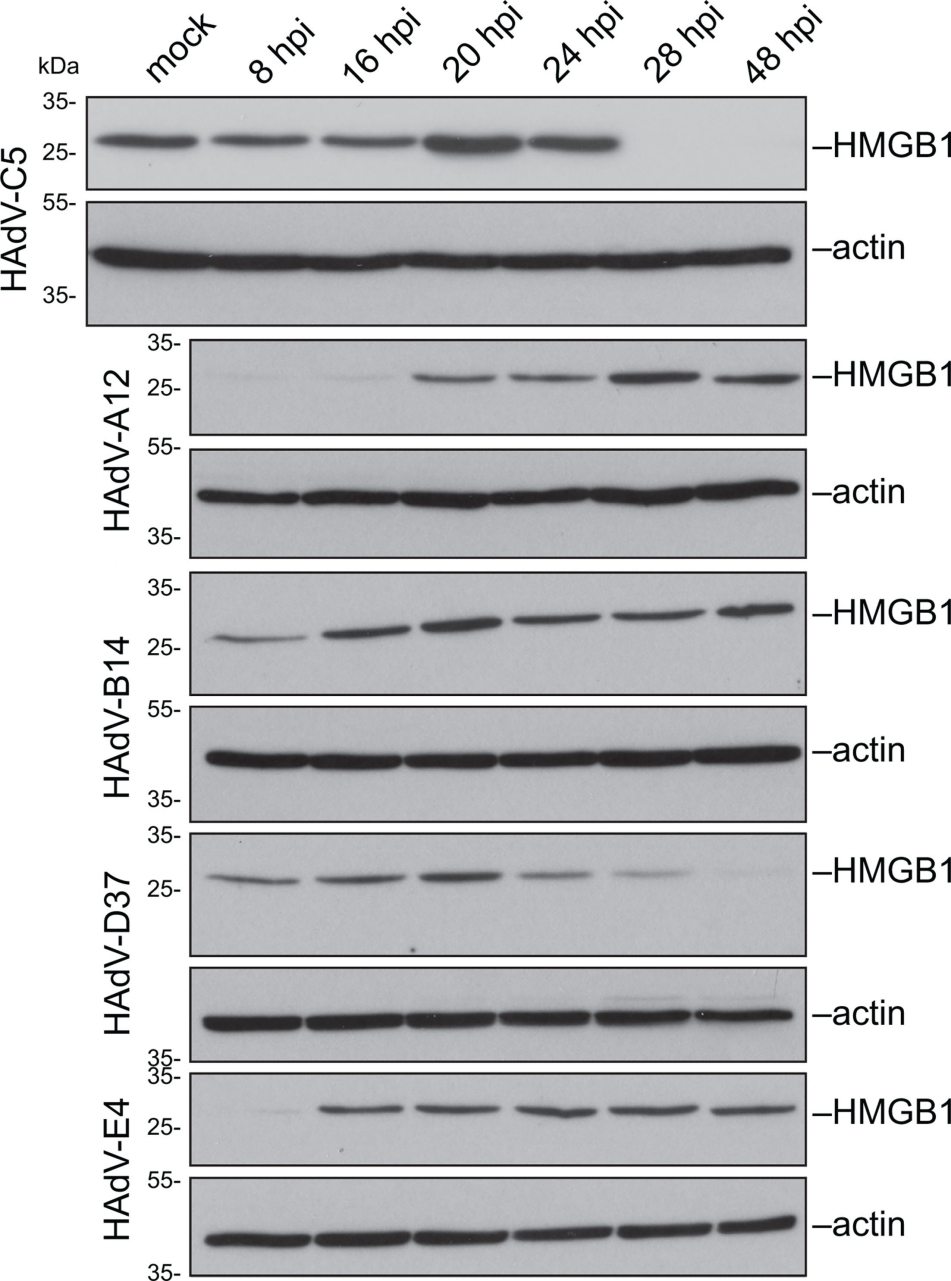
Downregulation of HMGB1 during HAdV infection is species-specific. HMGB1 steady-state levels from whole-cell lysates of A549 mock-infected or infected with HAdV-C5, -A12, -B14, -D37, or -E4 were harvested at 8, 16, 20, 24, 28, and 48 hpi, respectively, and analyzed by immunoblot to detect HMGB1. Actin was used as the loading control.

We previously demonstrated that HMGB1 localizes to RCs across multiple HAdV species (7). However, the impact of HMGB1 depletion on viral replication varies: in species C and D, HMGB1 knockdown results in a significant reduction in viral DNA levels, whereas in species B, it leads to a nearly fivefold increase (7). This suggests that, although HMGB1 relocalization to RCs is conserved, its functional role in viral replication is species-dependent. When we examined whether HMGB1 downregulation was conserved across different HAdV species, the Western blot analysis revealed that HMGB1 steady-state levels decrease late during HAdV-C5 and -D37 infections (Fig. 7), correlating with a pro-viral effect in these species, as previously reported (7).

Altogether, our findings demonstrate that HMGB1 is transcriptionally downregulated during HAdV-C5 infection, yet it plays a crucial role in viral DNA replication, gene expression, and progeny production. While HMGB1 relocalization to RCs is conserved across HAdV species, its functional impact on replication might be species-specific. Mechanistically, HMGB1 facilitates viral DNA synthesis through interactions with DBP in RCs. Our results highlight HMGB1 as a key host factor in HAdV infection and suggest that its regulation may be an important determinant of viral replication efficiency.

## DISCUSSION

HMGB1 localizes to HAdV RCs, facilitating viral DNA replication and transcription. Given its role in modulating chromatin dynamics and architecture, HMGB1 likely contributes to viral chromatin remodeling, enhancing the accessibility of viral promoters and thereby promoting efficient genome amplification and gene expression. Our findings suggest that HMGB1 regulation and function during HAdV infection are species-dependent, which is consistent with previous studies. For instance, HAdV-B7 infection leads to increased extracellular HMGB1 levels at late infection stages, and inhibition of HMGB1 reduces viral genome copy numbers, suggesting a pro-viral role in HAdV-B7 replication (31). Interestingly, HMGB1 knockdown reduced viral DNA levels in both HAdV-C5 and HAdV-D37 infections, and HMGB1 protein levels were downregulated in these two species, indicating a mechanistic link between HMGB1 depletion and viral replication efficiency. However, HAdV-D37, but not HAdV-C5, induced HMGB1 secretion, reinforcing species-specific differences in its regulation.

**Fig 8:**
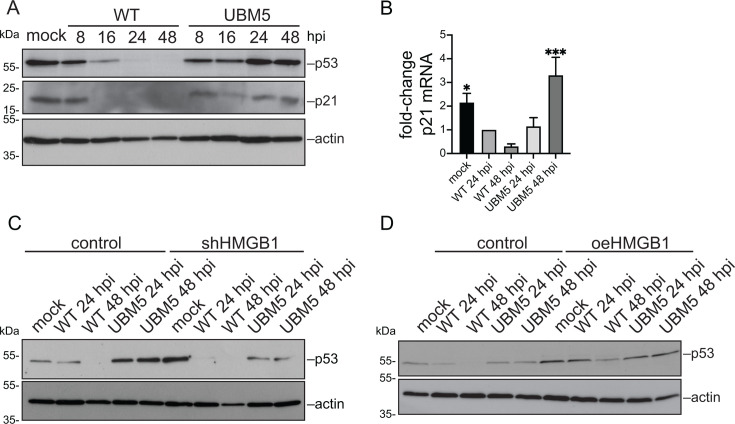
HMGB1 modulates the steady-state levels of p53 during HAdV-C5 infection. (**A**) Steady-state levels of p53 and p21 were measured in whole-cell lysates of A549 that were mock-infected or infected with HAdV-C5 WT or UBM5, harvested at 8, 16, 24, and 48 hpi and analyzed by immunoblotting. Actin was included as the loading control. (**B**) To measure the steady-state levels of the p21 mRNA, total RNA was isolated from A549 cells mock-infected or infected with HAdV-C5 WT or UBM5, harvested at 24 or 48 hpi. The RNA was reverse-transcribed and quantified by PCR. The plots show the fold-change values of the p21 mRNA, normalized to GAPDH mRNA and relative to the WT at 24 hpi. The error bars represent standard deviations. The data were analyzed using an ordinary one-way ANOVA test. *, *P* < 0.05; ***, *P* < 0.001. p53 protein levels were measured by immunoblotting at 24 and 48 hpi, in mock-infected, WT-, or UBM5-infected A549 cells, stably expressing the shRNA against HMGB1 (shHMGB1) (**C**) or overexpressing (oe) HMGB1 (**D**).

Our results reveal a complex interplay between viral infection and HMGB1. HMGB1 levels appear to be finely tuned—downregulated but not completely eliminated—since these low levels are necessary to optimize HAdV-C5 replication. This function of HMGB1 in HAdV infection is reminiscent of that of p53, whose levels are also decreased late during infection, despite promoting viral replication (32–35). While some studies suggest that p53 activity could be detrimental to viral infection, other reports indicate that it enhances HAdV late gene expression and replication, a role comparable to that of HMGB1 (32, 35). Moreover, the signaling pathways of both proteins are mutually interconnected. HMGB1 enhances binding of p53 to DNA (13), and its levels directly influence p53 expression (Fig. 8). The E3-ubiquitin ligase complex assembled by E1B-55K, responsible for targeting p53 for degradation during infection, is still functional in the UBM5 mutant virus (24). Nevertheless, in UBM5 infection, the levels of p53 remained stable or were even increased compared to mock- or WT-infected cells, paralleling the expression pattern of HMGB1. p21 mRNA levels were also increased in UBM5-infected cells (Fig. 8A and B). Notably, HMGB1 knockdown led to reduced p53 levels, whereas its overexpression increased them (Fig. 8C and D, respectively). These findings suggest that, in addition to DBP functions associated with RCs, DBP could also dictate how other restriction factors are controlled during infection. Further studies exploring the interaction between HMGB1 and p53 during HAdV infection could provide valuable insights into the mechanisms that regulate viral gene expression and DNA replication.

Our PLA experiments demonstrated that WT DBP interacts with HMGB1 (Fig. 1) and HMGB2 (data not shown). This interaction suggests that DBP may be responsible for recruiting these viral proteins to RCs. However, when RCs are not formed (as in UBM5 infection), DBP does not interact with these proteins. Therefore, these observations suggest a functional interaction between HMGB proteins and DBP on RC assembly, maintenance, or regulation of RC-associated activities. Further functional studies of the DBP interactome and understanding of the molecular mechanisms underlying the UBM5 phenotype would provide important insights into DBP and its role in RC-assembly and regulating virus-host interactions.

Because of the similarities between HMGB1 and HMGB2, one could expect them to have redundant functions during viral infection. To test this, we attempted a double knockdown of both proteins to determine whether viral replication would be more significantly reduced. However, the cells either did not survive or HMGB1 expression was restored. Nonetheless, single knockdown of HMGB2 had no significant effect on viral replication (data not shown), indicating that HMGB1 alone is responsible for this phenotype.

HMGB1 is involved in the life cycle of various viruses, including herpesviruses and influenza viruses, although its function varies (16). We, therefore, scanned the role of HMGB1 in other viral infections. In HSV-2 infection, HMGB1 transcription is decreased, yet the protein remains stable, whereas in influenza virus infection, HMGB1 binds viral ribonucleoproteins and promotes viral replication (25, 36). Although HMGB1 plays diverse roles during viral infections, it remains unclear whether its relocalization to RCs or other cellular compartments is a common feature across different viruses. Given the critical role of RCs in controlling the infected cell, it is important to consider their potential function in modulating HMGB1 activity. As discussed in the *Introduction*, restriction factors such as HMGB1 can be sequestered within RCs, either to subvert their antiviral functions or to be repurposed in favor of viral replication. Further investigation is needed to determine whether this mechanism is broadly applicable to other viral infections.

To further understand the role of HMGB1 in HAdV replication, future studies should focus on identifying functional domains that contribute to viral replication. Targeted mutagenesis could reveal key residues involved in DNA binding within RCs, protein-protein interactions with viral and cellular factors, and transcriptional regulation of viral genes. The HAdV core protein pVII has been shown to bind HMGB1 directly, reducing inflammation-induced HMGB1 secretion and neutrophil recruitment in mouse lungs, suggesting that HAdVs may actively modulate HMGB1 levels to evade immune responses (20). Further studies in *in vivo* models of HAdV infection could provide insights into the role of HMGB1 in viral pathogenesis and immune evasion, potentially leading to the development of novel antiviral strategies targeting HMGB1.

## MATERIALS AND METHODS

### Cell culture and virus propagation

The A549 and H1299 cell lines were maintained in monolayer cultures in Dulbecco’s modified Eagle’s medium (Gibco) supplemented with 10% fetal bovine serum (Gibco), 100 U/mL penicillin, and 100 µg/mL streptomycin (Gibco). Stable A549 and H1299 cell lines with either scrambled (scr) control or HMGB1 knockdown were generated using shRNAs, as previously described (7). The number of viable cells was determined using a Neubauer chamber and trypan blue dye, as described before (37). All cell lines were regularly tested for mycoplasma contamination.

We used H5pg4100 as the HAdV-C5 WT virus (38), along with the HAdV prototype strains HAdV-A12, HAdV-B14, HAdV-D37, and HAdV-E4 (39). The DBP mutant virus H5pm4251 (UBM5), which exhibits severe replication defects owing to a single-amino acid substitution in the essential DBP gene, has been described before (24). The mutant viruses H5*pm*4149 (E1B^-^) (40), H5*pm*4150 (E4orf3^-^) (41), and H5*pm*4154 (E4orf6^-^) (42) carry mutations in the open reading frames of E1B-55K, E4orf3, and E4orf6, respectively, and do not express the respective viral proteins. A multiplicity of infection (MOI) of 10 FFU/cell was used to infect all cell lines.

### Plasmids, transduction, and generation of stable cell lines

#### Stable HMGB1 overexpression and HMGB knockdown

A549 cells were seeded onto 6-well dishes and transduced with lentiviral particles. To overexpress HMGB1, lentiviral particles were produced with the plasmid RUSH reporter-HMGB1-SBP-GFP (Addgene, #172357), expressing HMGB1 under the CMV promoter and containing a puromycin resistance gene for cell selection. To prepare control cells, lentiviral particles were produced with a plasmid encoding an irrelevant sequence (target sequence: CCG GCA ACA AGA TGA AGA GCA CCA ACT CGA GTT GGT GCT CTT CAT CTT GTT GTT TTT; SHC002, Sigma-Aldrich) and a puromycin resistance gene for cell selection. The generation of the HMGB1 knockdown cells was published previously (24). Two days after transduction, fresh medium containing 2 µg/mL puromycin (Sigma-Aldrich) was added to select stably transduced cells. The cells were then propagated in fresh puromycin-containing medium. HMGB1 overexpression was verified by immunoblotting.

#### Transient HMGB1 transfection

To evaluate the effect of the overexpression of HMGB1 on viral proteins, H1299 cells were seeded in 10 cm dishes and transfected with 10 µg of the plasmid pcDNA3.1 Flag hHMGB1 (Addgene, #31609), expressing HMGB1 under the CMV promoter, using polyethylenimine (PEI) at a 3:1 ratio (PEI [µg]: total DNA [µg]), as previously described (3). At 12 h post-transfection, cells were mock-infected or infected with HAdV-C5 WT, harvested at 24 hpi, and processed for immunoblotting as described below.

#### Transient DBP transfection

To image DBP biomolecular condensates, H1299 cells were seeded on coverslips on 24-well plates and transfected with 1 µg of a plasmid encoding mCherry-DBP using polyethylenimine (PEI) at a 3:1 ratio (PEI [µg]: total DNA [µg]), as described before (3). The cells were fixed 24 h post-transfection and processed for immunofluorescence microscopy, as described below.

### Virus yield assay

To determine the virus yield, A549 cells stably overexpressing HMGB1 (oeHMGB1) were infected with HAdV-C5 and harvested at 48 hpi. Cells were lysed by three freeze-thaw cycles. The cell lysate supernatants were serially diluted in DMEM for infection of A549 cells, and the virus yield was determined using quantitative DBP immunofluorescence staining (DBP signal per cell) at 24 hpi, as described previously (43).

### Immunoblotting

To analyze steady-state protein concentrations, whole-cell lysates from mock-infected and HAdV-C5 WT- or UBM5-infected cells at the indicated time points were obtained. The protein concentration was measured spectrophotometrically using Bradford reagent (Bio-Rad). For immunoblotting, 50 µg of total protein was analyzed by SDS-PAGE, transferred onto nitrocellulose membranes (GE Healthcare), and blocked for 1 h at room temperature with 5% non-fat milk. Membranes were washed and incubated overnight at 4°C with the indicated primary antibodies (Table 1). After successive washes with PBS/0.1% Tween-20 (PBS-T), the membranes were incubated with secondary antibodies coupled to horseradish peroxidase (HRP) (Table 1) for at least 1 h at room temperature and thoroughly washed with PBS-T. Membranes were developed by enhanced chemiluminescence, as recommended by the manufacturer (Pierce, Thermo Scientific), on X-ray films (Fujifilm) or with a photodocumentator for chemiluminescence (ChemoStar**,** Intas Science Imaging).

**TABLE 1:** Antibodies^*a*^

	Antibody	Purpose	Dilution	Reference or source
Primary antibodies	α-DBP B6-8 mouse mAb	IF	1:5,000	(44)
	α-DBP rabbit pAb	IF and PLA	1:5,000	E. Tollefson, SLU (USA)
	α-HMGB1 W-18 mouse mAb	IF, WB, and PLA	1:200	Santa Cruz Biotechnology
	α-actin A-5441 mouse mAb	WB	1:10,000	Sigma-Aldrich
	α-IVa2 9F4 and 6C9 mouse mAb	IF	1:200	San Martín, CNB (Spain)
	α-p53 DO-1 mouse mAb	WB	1:1,000	Santa Cruz Biotechnology
	α-p21 (F-5) mouse mAb	WB	1:200	Santa Cruz Biotechnology
	α-capsid proteins rabbit pAb	WB	1:4,000	Abcam
	α-E1B M73 mouse mAb	WB	1:10	(45)
	α-E1B 2A6 mouse mAb	WB	1:10	(46)
Secondary antibodies	α-rabbit-HRP	WB	1:10,000	Jackson ImmunoResearch
	α-mouse-HRP	WB	1:10,000	Jackson ImmunoResearch
	α-mouse AlexaFluor 488	IF	1:1,500	Thermo Scientific
	α-rabbit AlexaFluor 488	IF	1:1,500	Thermo Scientific
	α-mouse AlexaFluor 555	IF	1:1,500	Thermo Scientific
	α-rabbit AlexaFluor 555	IF	1:1,500	Thermo Scientific
	α-mouse AlexaFluor 647	IF	1:1,500	Thermo Scientific

^*a*^ mAb, monoclonal antibody; pAb, polyclonal antibody; WB, Western (immuno-) blotting; IF, immunofluorescence microscopy; PLA, proximity ligation assay; HRP: horseradish peroxidase.

### Isolation and quantification of nucleic acids

Nucleic acids were isolated from total lysates of mock-infected, or HAdV-C5 WT-, or UBM5-infected cells. The QIAamp DNA Mini Kit (Qiagen) was used to isolate total DNA at 16 and 48 hpi following the manufacturer’s protocol. RNA was isolated at 24 and 48 hpi using the TRIzol protocol, as described previously (47). RNA was transcribed into cDNA using a reverse transcription system (Applied Biosystems), according to the manufacturer’s instructions, using random primers. DNA and cDNAs were quantified by real-time quantitative PCR (qPCR) using SYBR green reagents (SensiMix Plus SYBR; Quantace) and a Rotor-Gene cycler (Corbett Life Sciences). mRNA levels were normalized to GAPDH mRNA levels and relatively quantified using the ∆∆Ct method. The primers used are listed in Table 2.

**TABLE 2:** Primers

Primer name/target gene	Orientation	Sequence (5′–3′)
Viral DNA	Forward	GGT CTG GGC GTT AGG ATA CA
	Reverse	CAA TCA GTT TTC CGG CAA GT
E1A	Forward	GGT AGG TCT TGC AGG CTC CG
	Reverse	ATG AGG ACC TGT GGC ATG TTT G
E1B	Forward	GAG GGT AAC TCC AGG GTG CG
	Reverse	TTT CAC TAG CAT GAA GCA ACC ACA
IVa2	Forward	AGG GCG TCT CCA AGT TCT TCC
	Reverse	TGT TCC CAG CCA TAT CCC TCC
TPL	Forward	GCC TCC GAA CGG TAC TCC GCC
	Reverse	CGC CAC GGT GCT CAG CCT ACC
Hexon	Forward	CGC TGG ACA TGA CTT TTG AG
	Reverse	GAA CGG TGT GCG CAG GTA
HMGB1	Forward	AAC TTG TCG GGA GGA GCA TAA
	Reverse	CCA CCT CTC TGA GCA CTT CTT
p21	Forward	CTT GTA CCC TTG TGC CTC GCT
	Reverse	CGG ATT AGG GCT TCC TCT TGG
GAPDH	Forward	ACC ACA GTC CAT GCC ATC AC
	Reverse	TCC ACC ACC CTG TTG CTG TA

### Secretion of HMGB1

To measure HMGB1 secretion, we used the Lumit HMGB1 (Human/Mouse) Immunoassay (Promega) following the manufacturer’s instructions. The assays were performed using A549 cells, mock-infected or infected with HAdV-C5 WT or UBM5 viruses, and processed at 48 hpi. As positive controls, mock-infected cells were treated with 1 µM doxorubicin or 1 µM bortezomib to induce HMGB1 secretion. As a negative control, mock-infected cells were treated with 4 mM glycyrrhizin to block HMGB1 secretion. Luminescence was quantified using a Lumistar Omega microplate reader (BMG LABTECH).

### Promoter regulation/luciferase reporter assay

The HMGB1 promoter was amplified using the following primers: 5′ primer with an XhoI restriction site (ATA CTC GAG TTC AAT CCT TGA TGA CGT GTC CC) and 3′ primer with a HindIII restriction site (ATA AAG CTT TCC TGA CCC GCC GGC). The amplified sequence was purified and cloned into a pGL3-basic luciferase reporter vector (Promega). The sequence of the construct was verified by Sanger sequencing. The plasmid for the ML promoter was prepared as described before (48).

To evaluate activation of the HMGB1 promoter by dual luciferase reporter assays, subconfluent H1299 cells were treated with a transfection mixture of pTK-RL plasmid, ±HMGB1 promoter plasmid, and polyethylenimine (PEI) at a 3:1 ratio (PEI [µg]: total DNA [µg]), as previously described (48). At 12 h post-transfection, cells were mock-infected or infected with HAdV-C5 WT or UBM5 and harvested 24 and 48 hpi.

To measure activation of the ML promoter, shHMGB1 H1299 cells were treated with a transfection mixture of the pTK-RL plasmid, ±ML promoter plasmid, ±pcDNA3.1 Flag hHMGB1 (Addgene, #31609), expressing HMGB1 under the CMV promoter, and polyethylenimine (PEI) at a 3:1 ratio (PEI [µg]: total DNA [µg]), as previously described (48). At 12 h post-transfection, cells were mock-infected or infected with HAdV-C5 WT and harvested at 24 hpi.

HMGB1 or ML promoter activity was evaluated using lysed extracts on an automated luminometer (Berthold Technologies). All samples were normalized for transfection efficiency by measuring Renilla-luciferase activity from the cotransfected plasmid pTK-RL (Promega).

### Click chemistry, proximity ligation assay (PLA), and immunofluorescence microscopy

To visualize sites of active DNA replication, A549 cells were seeded on coverslips and mock-infected or infected with HAdV-C5 WT or UBM5. The Click-iT 5-ethynyl-2'-deoxyuridine (EdU) Cell Proliferation Kit for Imaging, Alexa Fluor 488 dye (Thermo Scientific) was used to label newly replicated DNA, according to the manufacturer’s instructions. EdU was added to the culture medium at 16 hpi, when an exponential increase in viral DNA synthesis in cell lines was observed, and cellular DNA replication was mostly abrogated. Cells were fixed at 24 hpi and processed, as described before (3).

PLAs were performed using A549 cells exactly as described before (7). Where indicated, cells were treated at 8 hpi with 4 mM glycyrrhizin to interfere with the DNA-binding activities of HMGB1 or with 100 ng/mL actinomycin D to inhibit DNA replication. The cells were fixed at 24 hpi.

The antibodies used are listed in Table 1. Coverslips were mounted on glass slides in Fluoroshield with 1,4-diazabicyclo [2.2.2] octane (Sigma-Aldrich). Cells were imaged using an inverted confocal scanning laser microscope (Nikon A1R HD25 equipped with a Nikon 60 x oil immersion NA 1.40 objective) or an inverted spinning disk microscope (Nikon Eclipse Ti-E stand with a Yokagawa CSU-W1 spinning disk confocal and a Nikon 100 x oil immersion NA 1.49 objective). Images were acquired as z-stacks spaced every 0.3 µm and processed using Fiji software (49). Image acquisition and processing were adjusted identically for the compared image sets. Figures are presented as maximum-intensity projections.

### Statistical analyses

Experiments were conducted with at least three biological replicates. Statistical analyses were performed using GraphPad Prism v9 (GraphPad Software), with details on specific tests provided in the corresponding figure legends. A *P*-value of ≤0.05 was considered statistically significant.

## References

[1] Tessier TM, Dodge MJ, MacNeil KM, Evans AM, Prusinkiewicz MA, Mymryk JS. 2021. Almost famous: human adenoviruses (and what they have taught us about cancer). Tumour Virus Res 12:200225. doi:10.1016/j.tvr.2021.200225 34500123 PMC8449131

[2] Hidalgo P, Gonzalez RA. 2019. Formation of adenovirus DNA replication compartments. FEBS Lett 593:3518–3530. doi:10.1002/1873-3468.13672 31710378

[3] Hidalgo Paloma, Pimentel A, Mojica-Santamaría D, von Stromberg K, Hofmann-Sieber H, Lona-Arrona C, Dobner T, González RA. 2021. Evidence that the adenovirus single-stranded dna binding protein mediates the assembly of biomolecular condensates to form viral replication compartments. Viruses 13:1778. doi:10.3390/v13091778 34578359 PMC8473285

[4] Charman M, Herrmann C, Weitzman MD. 2019. Viral and cellular interactions during adenovirus DNA replication. FEBS Lett 593:3531–3550. doi:10.1002/1873-3468.13695 31764999 PMC6928415

[5] Charman M, Weitzman MD. 2020. Replication compartments of DNA viruses in the nucleus: location, location, location. Viruses 12:151. doi:10.3390/v12020151 32013091 PMC7077188

[6] Schmid M, Speiseder T, Dobner T, Gonzalez RA. 2014. DNA virus replication compartments. J Virol 88:1404–1420. doi:10.1128/JVI.02046-13 24257611 PMC3911613

[7] Hidalgo Paloma, Torres A, Jean Beltran PM, López-Leal G, Bertzbach LD, Dobner T, Flint SJ, Cristea IM, González RA. 2025. The protein composition of human adenovirus replication compartments. mBio 16:e0214424. doi:10.1128/mbio.02144-24 39611842 PMC11708036

[8] Hidalgo P, Anzures L, Hernández-Mendoza A, Guerrero A, Wood CD, Valdés M, Dobner T, Gonzalez RA. 2016. Morphological, biochemical, and functional study of viral replication compartments isolated from adenovirus-infected cells. J Virol 90:3411–3427. doi:10.1128/JVI.00033-16 26764008 PMC4794690

[9] Hidalgo P, Gonzalez RA. 2015. Isolation of viral replication compartment-enriched sub-nuclear fractions from adenovirus-infected normal human cells. J Vis Exp. doi:10.3791/53296:53296 PMC4692707 26649626

[10] Chikhirzhina E, Starkova T, Beljajev A, Polyanichko A, Tomilin A. 2020. Functional diversity of non-histone chromosomal protein HMGB1. Int J Mol Sci 21:7948. doi:10.3390/ijms21217948 33114717 PMC7662367

[11] Starkova T, Polyanichko A, Tomilin AN, Chikhirzhina E. 2023. Structure and functions of HMGB2 protein. Int J Mol Sci 24:8334. doi:10.3390/ijms24098334 37176041 PMC10179549

[12] Starkova TY, Polyanichko AM, Artamonova TO, Tsimokha AS, Tomilin AN, Chikhirzhina EV. 2023. Structural characteristics of high-mobility group proteins HMGB1 and HMGB2 and their interaction with DNA. Int J Mol Sci 24:3577. doi:10.3390/ijms24043577 36834988 PMC9962726

[13] Chen R, Kang R, Tang D. 2022. The mechanism of HMGB1 secretion and release. Exp Mol Med 54:91–102. doi:10.1038/s12276-022-00736-w 35217834 PMC8894452

[14] Kang R, Chen R, Zhang Q, Hou W, Wu S, Cao L, Huang J, Yu Y, Fan X, Yan Z, Sun X, Wang H, Wang Q, Tsung A, Billiar TR, Zeh HJ III, Lotze MT, Tang D. 2014. HMGB1 in health and disease. Mol Aspects Med 40:1–116. doi:10.1016/j.mam.2014.05.001 25010388 PMC4254084

[15] Yuan J, Guo L, Ma J, Zhang H, Xiao M, Li N, Gong H, Yan M. 2024. HMGB1 as an extracellular pro-inflammatory cytokine: implications for drug-induced organic damage. Cell Biol Toxicol 40:55. doi:10.1007/s10565-024-09893-2 39008169 PMC11249443

[16] Ding X, Li S, Zhu L. 2021. Potential effects of HMGB1 on viral replication and virus infection-induced inflammatory responses: a promising therapeutic target for virus infection-induced inflammatory diseases. Cytokine Growth Factor Rev 62:54–61. doi:10.1016/j.cytogfr.2021.08.003 34503914

[17] Watt F, Molloy PL. 1988. High mobility group proteins 1 and 2 stimulate binding of a specific transcription factor to the adenovirus major late promoter. Nucleic Acids Res 16:1471–1486. doi:10.1093/nar/16.4.1471 2831501 PMC336328

[18] Lynch KL, Dillon MR, Bat-Erdene M, Lewis HC, Kaai RJ, Arnold EA, Avgousti DC. 2021. A viral histone-like protein exploits antagonism between linker histones and HMGB proteins to obstruct the cell cycle. Curr Biol 31:5227–5237. doi:10.1016/j.cub.2021.09.050 34666003 PMC8665055

[19] Arnold EA, Kaai RJ, Leung K, Brinkley MR, Kelnhofer-Millevolte LE, Guo MS, Avgousti DC. 2023. Adenovirus protein VII binds the a-box of HMGB1 to repress interferon responses. PLoS Pathog 19:e1011633. doi:10.1371/journal.ppat.1011633 37703278 PMC10519595

[20] Avgousti DC, Herrmann C, Kulej K, Pancholi NJ, Sekulic N, Petrescu J, Molden RC, Blumenthal D, Paris AJ, Reyes ED, Ostapchuk P, Hearing P, Seeholzer SH, Worthen GS, Black BE, Garcia BA, Weitzman MD. 2016. A core viral protein binds host nucleosomes to sequester immune danger signals. Nature 535:173–177. doi:10.1038/nature18317 27362237 PMC4950998

[21] Darweesh M, Kamel W, Gavrilin MA, Akusjärvi G, Svensson C. 2019. Adenovirus VA RNAI blocks ASC oligomerization and inhibits NLRP3 inflammasome activation. Front Immunol 10:2791. doi:10.3389/fimmu.2019.02791 31849970 PMC6901988

[22] Saha A, Islam MM, Kumar R, Ismail AM, Garcia E, Gullapali RR, Chodosh J, Rajaiya J. 2025. Virus and cell specific HMGB1 secretion and subepithelial infiltrate formation in adenovirus keratitis. PLoS Pathog 21:e1013184. doi:10.1371/journal.ppat.1013184 40367285 PMC12101768

[23] Bertzbach LD, Seddar L, von Stromberg K, Ip W-H, Dobner T, Hidalgo P. 2024. The adenovirus DNA-binding protein DBP. J Virol 98:e0188523. doi:10.1128/jvi.01885-23 38197632 PMC10878046

[24] Boddin J, Ip W-H, Wilkens B, von Stromberg K, Ching W, Koyuncu E, Bertzbach LD, Dobner T. 2022. A single amino acid switch in the adenoviral DNA binding protein abrogates replication center formation and productive viral infection. mBio 13:e0014422. doi:10.1128/mbio.00144-22 35254132 PMC9040859

[25] Moisy D, Avilov SV, Jacob Y, Laoide BM, Ge X, Baudin F, Naffakh N, Jestin JL. 2012. HMGB1 protein binds to influenza virus nucleoprotein and promotes viral replication. J Virol 86:9122–9133. doi:10.1128/JVI.00789-12 22696656 PMC3416134

[26] Mollica L, De Marchis F, Spitaleri A, Dallacosta C, Pennacchini D, Zamai M, Agresti A, Trisciuoglio L, Musco G, Bianchi ME. 2007. Glycyrrhizin binds to high-mobility group box 1 protein and inhibits its cytokine activities. Chem Biol 14:431–441. doi:10.1016/j.chembiol.2007.03.007 17462578

[27] Ip WH, Tatham MH, Krohne S, Gruhne J, Melling M, Meyer T, Gornott B, Bertzbach LD, Hay RT, Rodriguez E, Dobner T. 2023. Adenovirus E1B-55K controls SUMO-dependent degradation of antiviral cellular restriction factors. J Virol 97:e0079123. doi:10.1128/jvi.00791-23 37916833 PMC10688335

[28] Pennella MA, Liu Y, Woo JL, Kim CA, Berk AJ. 2010. Adenovirus E1B 55-kilodalton protein is a p53-SUMO1 E3 ligase that represses p53 and stimulates its nuclear export through interactions with promyelocytic leukemia nuclear bodies. J Virol 84:12210–12225. doi:10.1128/JVI.01442-10 20861261 PMC2976411

[29] Sohn SY, Hearing P. 2016. The adenovirus E4-ORF3 protein functions as a SUMO E3 ligase for TIF-1γ sumoylation and poly-SUMO chain elongation. Proc Natl Acad Sci USA 113:6725–6730. doi:10.1073/pnas.1603872113 27247387 PMC4914182

[30] Cheng CY, Gilson T, Dallaire F, Ketner G, Branton PE, Blanchette P. 2011. The E4orf6/E1B55K E3 ubiquitin ligase complexes of human adenoviruses exhibit heterogeneity in composition and substrate specificity. J Virol 85:765–775. doi:10.1128/JVI.01890-10 21068234 PMC3020000

[31] Tang Z, Zang N, Fu Y, Ye Z, Chen S, Mo S, Ren L, Liu E. 2018. HMGB1 mediates HAdV-7 infection-induced pulmonary inflammation in mice. Biochem Biophys Res Commun 501:1–8. doi:10.1016/j.bbrc.2018.03.145 29571731

[32] Royds JA, Hibma M, Dix BR, Hananeia L, Russell IA, Wiles A, Wynford-Thomas D, Braithwaite AW. 2006. P53 promotes adenoviral replication and increases late viral gene expression. Oncogene 25:1509–1520. doi:10.1038/sj.onc.1209185 16247442

[33] Yew PR, Berk AJ. 1992. Inhibition of p53 transactivation required for transformation by adenovirus early 1B protein. Nature 357:82–85. doi:10.1038/357082a0 1533443

[34] Wright J, Leppard KN. 2013. The human adenovirus 5 L4 promoter is activated by cellular stress response protein p53. J Virol 87:11617–11625. doi:10.1128/JVI.01924-13 23966406 PMC3807333

[35] Hall AR, Dix BR, O’Carroll SJ, Braithwaite AW. 1998. P53-dependent cell death/apoptosis is required for a productive adenovirus infection. Nat Med 4:1068–1072. doi:10.1038/2057 9734403

[36] Borde C, Barnay-Verdier S, Gaillard C, Hocini H, Maréchal V, Gozlan J. 2011. Stepwise release of biologically active HMGB1 during HSV-2 infection. PLoS One 6:e16145. doi:10.1371/journal.pone.0016145 21283827 PMC3023802

[37] Ching W, Koyuncu E, Singh S, Arbelo-Roman C, Hartl B, Kremmer E, Speiseder T, Meier C, Dobner T. 2013. A ubiquitin-specific protease possesses a decisive role for adenovirus replication and oncogene-mediated transformation. PLoS Pathog 9:e1003273. doi:10.1371/journal.ppat.1003273 23555268 PMC3610741

[38] Groitl P, Dobner T. 2007. Construction of adenovirus type 5 early region 1 and 4 virus mutants. Methods Mol Med 130:29–39. doi:10.1385/1-59745-166-5:29 17401162

[39] Kosulin K, Lam E, Heim A, Dobner T, Rodríguez E. 2018. Broad-Spectrum Antiviral Activity of the Deubiquitinase Inhibitor HBX against Human Adenoviruses. Antivir Ther (Lond) 23:475–483. doi:10.3851/IMP3230 29557344

[40] Kindsmüller K, Schreiner S, Leinenkugel F, Groitl P, Kremmer E, Dobner T. 2009. A 49-kilodalton isoform of the adenovirus type 5 early region 1B 55-kilodalton protein is sufficient to support virus replication. J Virol 83:9045–9056. doi:10.1128/JVI.00728-09 19587039 PMC2738261

[41] Forrester NA, Patel RN, Speiseder T, Groitl P, Sedgwick GG, Shimwell NJ, Seed RI, Catnaigh PÓ, McCabe CJ, Stewart GS, Dobner T, Grand RJA, Martin A, Turnell AS. 2012. Adenovirus E4orf3 targets transcriptional intermediary factor 1γ for proteasome-dependent degradation during infection. J Virol 86:3167–3179. doi:10.1128/JVI.06583-11 22205733 PMC3302322

[42] Blanchette P, Kindsmüller K, Groitl P, Dallaire F, Speiseder T, Branton PE, Dobner T. 2008. Control of mRNA export by adenovirus E4orf6 and E1B55K proteins during productive infection requires E4orf6 ubiquitin ligase activity. J Virol 82:2642–2651. doi:10.1128/JVI.02309-07 18184699 PMC2258987

[43] Ching W, Dobner T, Koyuncu E. 2012. The human adenovirus type 5 E1B 55-kilodalton protein is phosphorylated by protein kinase CK2. J Virol 86:2400–2415. doi:10.1128/JVI.06066-11 22190719 PMC3302271

[44] Reich NC, Sarnow P, Duprey E, Levine AJ. 1983. Monoclonal antibodies which recognize native and denatured forms of the adenovirus DNA-binding protein. Virology (Auckl) 128:480–484. doi:10.1016/0042-6822(83)90274-x 6310869

[45] Harlow E, Franza BR, Schley C. 1985. Monoclonal antibodies specific for adenovirus early region 1A proteins: extensive heterogeneity in early region 1A products. J Virol 55:533–546. doi:10.1128/JVI.55.3.533-546.1985 3894685 PMC255001

[46] Sarnow P, Sullivan CA, Levine AJ. 1982. A monoclonal antibody detecting the adenovirus type 5-E1b-58Kd tumor antigen: characterization of the E1b-58Kd tumor antigen in adenovirus-infected and -transformed cells. Virology (Auckl) 120:510–517. doi:10.1016/0042-6822(82)90054-x 7048730

[47] Ip WH, Wilkens B, Solomatina A, Martin J, Melling M, Hidalgo P, Bertzbach LD, Speiseder T, Dobner T. 2021. Differential Regulation of Cellular FAM111B by Human Adenovirus C Type 5 E1 Oncogenes. Viruses 13:1015. doi:10.3390/v13061015 34071532 PMC8227810

[48] Schreiner S, Bürck C, Glass M, Groitl P, Wimmer P, Kinkley S, Mund A, Everett RD, Dobner T. 2013. Control of human adenovirus type 5 gene expression by cellular Daxx/ATRX chromatin-associated complexes. Nucleic Acids Res 41:3532–3550. doi:10.1093/nar/gkt064 23396441 PMC3616723

[49] Schindelin J, Arganda-Carreras I, Frise E, Kaynig V, Longair M, Pietzsch T, Preibisch S, Rueden C, Saalfeld S, Schmid B, Tinevez JY, White DJ, Hartenstein V, Eliceiri K, Tomancak P, Cardona A. 2012. Fiji: an open-source platform for biological-image analysis. Nat Methods 9:676–682. doi:10.1038/nmeth.2019 22743772 PMC3855844

